# Supplementary material for: EnzML: multi-label prediction of enzyme classes using InterPro signatures
Source: BMC Bioinformatics. 2012 Apr 25;13:61. doi: 10.1186/1471-2105-13-61 (PMC3483700; doi:10.1186/1471-2105-13-61)
Supplement: Addtional file 5 — The Java code to format the data files, evaluate and predict. The file enzml_java_code.tar.gz contains the Java code used to format database data to ARFF and XML formats, to execute cross and train-test (jackknife) evaluations and to record evaluation results to database. More information is included in the readme.txt file and the Javadoc files. The code can be used with a MySQL database. To use a different database software, other JDBC drivers might be required. [file 1471-2105-13-61-S5.gz › java_code/enzml2011/doc/uk/ac/ed/inf/enzml/mulan/learn/traintest/TrainTestFullRun.html]

TrainTestFullRun


---


|  |  |  |
| --- | --- | --- |
| **Overview** \| **Package** \| **Class** \| **Use** \| **Tree** \| **Deprecated** \| **Index** \| **Help** | |  |
| **PREV CLASS**   NEXT CLASS | **FRAMES**    **NO FRAMES**     **All Classes** |  |
| SUMMARY: NESTED \| FIELD \| CONSTR \| METHOD | DETAIL: FIELD \| CONSTR \| METHOD |  |


---


## uk.ac.ed.inf.enzml.mulan.learn.traintest Class TrainTestFullRun

```
java.lang.Object
  uk.ac.ed.inf.enzml.mulan.learn.traintest.TrainTestFullRun
```

---

``` public class TrainTestFullRun extends java.lang.Object ```

Runs a full train/test evaluation: 1. generate the ARFF file for training and
the (old) file for testing 2. filter the (old) test arff file by the train
file 3. generate a .model file containing the serialised classifier trained
on the train arff 4. train on the train arff and test on the (new, filtered)
test arff

**Version:**
:   28 Mar 2011

**Author:**
:   Luna De Ferrari luna.deferrari-at-ed.ac.uk

---

**Constructor Summary**

`TrainTestFullRun()`


| **Method Summary** | |
| --- | --- |
| `static void` | `fullRun()`             Executes: 1. |
| `static void` | `main(java.lang.String[] args)` |
| `static void` | `run2_3()`             Executes 2. |
| `static void` | `run3()`             When files and trained model are already available, it executes exclusively the train-test evaluation |

**Methods inherited from class java.lang.Object**

`equals, getClass, hashCode, notify, notifyAll, toString, wait, wait, wait`

**Constructor Detail**

### TrainTestFullRun

```
public TrainTestFullRun()
```


**Method Detail**

### fullRun

```
public static void fullRun()
```

:   Executes: 1. filtering the test file by the train file, 2. model training
    and serialisation, 3. train-test evaluation

---


### main

```
public static void main(java.lang.String[] args)
```

---


### run2\_3

```
public static void run2_3()
```

:   Executes 2. model training and serialisation, and 3. train-test
    evaluation (but not filtering the test file by the train file, that must
    already be available by id)

---


### run3

```
public static void run3()
```

:   When files and trained model are already available, it executes
    exclusively the train-test evaluation


---


|  |  |  |
| --- | --- | --- |
| **Overview** \| **Package** \| **Class** \| **Use** \| **Tree** \| **Deprecated** \| **Index** \| **Help** | |  |
| **PREV CLASS**   NEXT CLASS | **FRAMES**    **NO FRAMES**     **All Classes** |  |
| SUMMARY: NESTED \| FIELD \| CONSTR \| METHOD | DETAIL: FIELD \| CONSTR \| METHOD |  |


---
